# Mesenchymal Stromal/Stem Cells in Regenerative Medicine and Tissue Engineering

**DOI:** 10.1155/2018/8031718

**Published:** 2018-08-19

**Authors:** Ross E. B. Fitzsimmons, Matthew S. Mazurek, Agnes Soos, Craig A. Simmons

**Affiliations:** ^1^Institute of Biomaterials and Biomedical Engineering, University of Toronto, 164 College Street, Toronto, ON, Canada M5S 3G9; ^2^Translational Biology and Engineering Program, Ted Rogers Centre for Heart Research, 661 University Ave, Toronto, ON, Canada M5G 1M1; ^3^Division of Gastroenterology and Hepatology, Department of Medicine, University of Calgary, Calgary, AB, Canada T2N 4Z6; ^4^Department of Mechanical and Industrial Engineering, University of Toronto, 5 King's College Road, Toronto, ON, Canada M5S 3G8

## Abstract

As a result of over five decades of investigation, mesenchymal stromal/stem cells (MSCs) have emerged as a versatile and frequently utilized cell source in the fields of regenerative medicine and tissue engineering. In this review, we summarize the history of MSC research from the initial discovery of their multipotency to the more recent recognition of their perivascular identity *in vivo* and their extraordinary capacity for immunomodulation and angiogenic signaling. As well, we discuss long-standing questions regarding their developmental origins and their capacity for differentiation toward a range of cell lineages. We also highlight important considerations and potential risks involved with their isolation, ex vivo expansion, and clinical use. Overall, this review aims to serve as an overview of the breadth of research that has demonstrated the utility of MSCs in a wide range of clinical contexts and continues to unravel the mechanisms by which these cells exert their therapeutic effects.

## 1. Introduction

**Figure 1:**
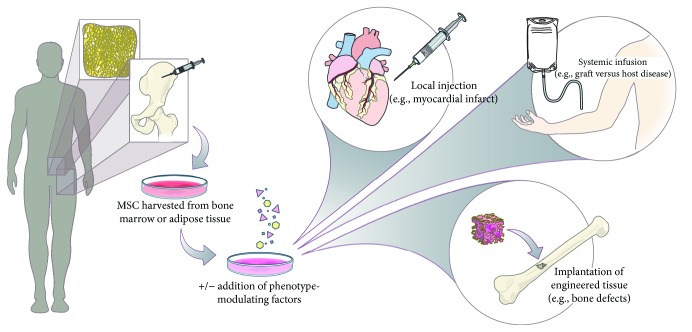
Strategies for mesenchymal stromal/stem cell- (MSC-) based therapies. MSCs may be isolated from a number of tissues (e.g., bone marrow, adipose tissue, and umbilical cord) and optionally cultured prior to clinical use. Depending on the specific application, MSC suspensions may then be introduced intravenously or by local injection to achieve the desired therapeutic effects, such as treating autoimmune diseases or stimulating local tissue repair and vascularization, respectively. MSCs may also be utilized for engineering tissues by first promoting their differentiation toward a desired cell type (e.g., osteoblasts, chondrocytes, and adipocytes) prior to being surgically implanted, often along with scaffold material.

By merit of their regenerative secretome and their capacity for differentiation toward multiple mesenchymal lineages, the fibroblastic cell type termed mesenchymal stromal/stem cells (MSCs) shows promise for a wide range of tissue engineering and regenerative medicine applications (Figure 1). As a result of their therapeutic versatility and the multitude of promising clinical results thus far, MSCs are poised to become an increasingly significant cell source for regenerative therapies as medicine evolves to focus on personalized and cell-based therapeutics. Given their emerging importance, this review aims to provide an overview of historical and ongoing work aimed at understanding and better utilizing these cells for therapeutic purposes.

## 2. Initial Discoveries and the Evolving Definition of “MSC”

The initial discovery of MSCs is attributed to Friedenstein et al. who discovered a fibroblastic cell type derived from mouse and guinea pig bone marrow that could produce clonal colonies capable of generating bone and reticular tissue when heterotopically transplanted [1, 2]. The subsequent discovery that colonies of this cell type can generate cartilage and adipose tissue, in addition to bone, gave rise to the descriptor *mesenchymal stem cells*, as originally coined by Arnold Caplan [3]. Finally, Pittenger et al. established that human bone marrow also contains a subpopulation of stromal cells that are genuinely multipotent stem cells by demonstrating single colonies have trilineage mesenchymal potential [4].

Over time, the acronym MSC has come to take on multiple meanings including, mesenchymal stem cell, mesenchymal stromal cell, and multipotent stromal cell. To help clarify this, the International Society for Cellular Therapy (ISCT) has officially defined MSCs as *multipotent mesenchymal stromal cells* and suggests this to mean the plastic-adherent fraction from stromal tissues, while reserving the term *mesenchymal stem cells* to mean the subpopulation that actually has the two cardinal stem cell properties (*i.e.*, self-renewal and the capacity to differentiate down multiple lineages) [5]. Furthermore, ISCT has also defined MSCs as meeting several criteria including (i) being plastic adherent, (ii) having osteogenic, adipogenic, and chondrogenic trilineage differentiation potential, (iii) and being positive (>95%) and negative (<2%) for a panel of cell surface antigens. Positive markers for human MSCs include CD73 (also present on lymphocytes, endothelial cells, smooth muscle cells, and fibroblasts), CD90 (also present on hematopoietic stem cells, lymphocytes, endothelial cells, neurons, and fibroblasts), and CD105 (also found on endothelial cells, monocytes, hematopoietic progenitors, and fibroblasts) [6]. Negative markers include CD34 (present on hematopoietic progenitors and endothelial cells), CD45 (a pan-leukocyte marker), CD14 or CD11b (present on monocytes and macrophages), CD79-*α* or CD19 (present on B cells), and HLA-DR unless stimulated with IFN-*γ* (present on macrophages, B cells, and dendritic cells) [5]. It should be noted, however, that the validity of CD34 as a negative marker has recently been called into question and may require reexamination [6, 7].

As these elaborate inclusionary and exclusionary criteria highlight, no single MSC-specific epitope has been discovered, unlike for some other stem cell populations (e.g., LGR5, which labels resident stem cells in hair follicles and intestinal crypts) [8, 9]. However, some markers may be used to enrich for the stem cell population, including Stro-1, CD146, CD106, CD271, MSCA-1, and others (Table 1) [6, 10–13]. This unfortunate lack of a single definitive marker continues to confound the interpretation of a broad range of studies given that sorting out the canonical MSC population from the adherent fraction is rarely done, leading to the perennial question of which subpopulation in the adherent stromal fraction is actually eliciting the observed effects. This lack of a definitive MSC marker has also contributed to the challenge of delineating the exact *in vivo* location, function, and developmental origin of MSCs.

## 3. MSC Adult Anatomical Location

In the bone marrow, where MSCs were first discovered, MSCs have been reported to typically localize near the sinusoidal endothelium in close association with the resident hematopoietic stem cells (HSCs) [14, 15]. In addition to serving as osteogenic progenitors, such MSCs have been shown to play an important role in regulating HSC function by maintaining the HSC niche and by secreting trophic factors such as angiopoietin 1 (Ang1), stem cell factor (SCF), and CXC ligand 12 (CXCL12) [10]. Beyond the bone marrow, MSC/MSC-like populations have also been found in many adult tissues (*e.g.*, skin, pancreas, heart, brain, lung, kidney, adipose tissue, cartilage, and tendon) [16–19]. Such a broad anatomical distribution would suggest a common and ubiquitous MSC niche exists throughout the body. Indeed, evidence suggests that many MSC populations are specifically located near blood vessels and are in fact a subpopulation of pericytes that reside on capillaries and venules [20]. Supporting observations include the fact that pericytes and MSCs express similar surface antigens, and that cells in perivascular positions were found to express MSC markers in human bone marrow and dental pulp [16, 21]. Perhaps most definitively, Crisan et al. found that cells positive for NG2, CD146, and PDGFR-*β* specifically stained pericytes in multiple human tissues, and when cells with these markers were isolated, they were shown to have trilineage potential *in vitro* and were osteogenic once transplanted *in vivo* [22]. The converse, that all pericytes are MSCs, is not thought to be the case [20].

In addition to being abluminal to microvessels, it should be noted that a Gli1^+^ MSC-like population has also been found to reside within the adventitia of larger vessels in mice. The Gli1^+^ population exhibits trilineage differentiation *in vitro* and is thought to play a role in arterial calcification *in vivo* [23–25]. Similarly, a MSC population with a CD34^+^ CD31^−^ CD146^−^ CD45^−^ phenotype has been discovered to reside within the adventitia of human arteries and veins suggesting that not all perivascular MSCs are pericyte-like cells in humans [7]. Furthermore, a MSC population has also been isolated from the perivascular tissue of umbilical cords (human umbilical cord perivascular cells (HUCPVCs)) which shows promise for tissue engineering applications given the cells' noninvasive extraction and their relatively high abundance and proliferative capacity, compared to bone marrow-derived MSCs [26–28].

Finally, despite the prevalent view that MSCs reside in perivascular niches, some MSC populations may reside in avascular regions as well. For example, a lineage tracing study focused on murine tooth repair demonstrated that while some odontoblasts descend from cells expressing the pericyte marker, NG2, the majority of odontoblasts did not, suggestive of a nonpericyte origin (or at least not from NG2-positive pericytes) [29]. Additionally, MSCs have been isolated from tissues that are typically avascular, including human synovial tissue [30–32] and porcine aortic valve [33]. However, there are fenestrated capillaries localized near the synovial surface [34], and diseased sclerotic and stenotic valves can be partially vascularized [35, 36], raising the possibility of MSCs trafficking from one anatomical location to another (*e.g.*, synovium-associated vasculature to avascular cartilage) and innate differences in the local presence or absence of perivascular MSCs. Future work focused on these questions will have important implications for understanding disease progression and potential regenerative avenues.

## 4. MSC Developmental Origins

Presently, there are considered to be multiple developmental origins of MSCs. Unsurprisingly, given their mesenchymal differentiation potential, certain subsets of MSCs are derived from mesodermal precursors, such as lateral plate mesoderm- (LPM-) derived mesoangioblast cells from the embryonic dorsal aorta [37, 38]. Support for this comes from the observation that mesoangioblast cells isolated from the mouse dorsal aorta and then grafted into chick embryos incorporated into several mesodermal tissues (bone, cartilage, muscle, and blood) [39].

Other reports suggest MSCs partly descend from a subpopulation of neural crest cells, with the remaining MSCs descending from unknown origins. Support for this comes from the observation that a population of murine Sox1^+^ trunk neuroepithelial cells could undergo clonogenic expansion and maintain adipogenic, chondrogenic, and osteogenic differentiation *in vitro* [40]. This neural crest origin may help explain why MSCs have neural differentiation potential and why human bone marrow-derived MSCs can be enriched for using antibodies against nerve growth factor receptor [12, 38]. Given their lineage tracing results, the authors claimed that neural crest-derived MSCs are the earliest MSCs to arise in the embryo, but they did note that other MSCs must also arise later on in development as not all MSCs detected were found to be of a neural crest origin. Corroborating this, a lineage tracing study using the promoter from Protein-0, a neural crest-associated marker, found that only a portion of bone marrow-derived MSCs were labeled in adult mice, suggestive of both a neural crest and nonneural crest origin [41].

It is possible that the indefinite nonneural crest source of MSCs observed in these studies may be mesoangioblasts or another mesoderm-derived cell type. It has also been suggested that data indicative of a mesoangioblast origin may alternatively be explained by simply “contamination” of neural crest cells as the neural tube is close to dorsal aorta at day 9.5 [38]. With regard to human MSC origins, similar dual mesoderm and neural crest origins may also exist given that human iPSCs differentiated toward these two lineages can both give rise to MSC-like cells [42, 43]. Further study will be required to resolve these issues and to elucidate if any lasting functional dissimilarities exist between MSC subpopulations that arise from differing time periods and locations during development.

## 5. MSC Expansion in Culture

Once isolated from their respective *in vivo* locations, human MSC populations can be expanded up to several hundredfold while maintaining their multipotency and capacity to form fibroblastic colony-forming units (CFU-F) provided the cells are seeded at a satisfactorily low seeding density (~10–100 cell/cm^2^) [44]. When cultured at low clonal density, MSCs take on a highly proliferative phenotype and maintain their trilineage potential; such cells have become commonly referred to as RS-MSCs (rapidly self-renewing MSCs). This proliferative phase is thought to be dependent on Dickkopf-related protein 1 (Dkk-1) autocrine signaling which inhibits Wnt signaling that would otherwise promote differentiation [45]. Favorable for minimizing risk to patients, *in vitro* proliferation of human MSCs exhibits a relatively low frequency of oncogenic transformation (<10^−9^) [46–48]. This is in stark contrast with murine MSCs which frequently gain chromosomal defects *in vitro* and often produce fibrosarcomas when injected back into mice [49].

With time, sparsely plated human MSCs create colonies with distinct *in vitro* niches with the inner cells expressing differentiation markers and the outer cells exhibiting a more RS-MSC phenotype with high motility and proliferation [50, 51]. Yet, when replated, both inner and outer regions create colonies similar to the original, implying differentiation of the inner colony is reversible to some extent [51]. If MSCs are seeded at a higher density (~1000 cell/cm^2^) and/or are cultured to confluence, RS-MSCs will decrease and SR-MSCs (slowly replicating MSCs) will increase over time, while both the CFU-F and proportion of multipotent cells will gradually decline [44, 51]. This dynamic nature during culture underlines the importance of properly maintaining MSC cultures to ensure maximum self-renewal and the maintenance of differentiation potential for downstream applications.

## 6. MSC Differentiation Potential

As mentioned earlier, by definition, MSCs have trilineage potential with the capacity to undergo osteogenesis, adipogenesis, and chondrogenesis contingent on their exposure to the particular soluble factors in their microenvironment. Differentiation protocols for driving differentiation toward these lineages have been routinely utilized and extensively optimized [52, 53]. For example, osteogenesis typically involves the use of dexamethasone, *β*-glycerolphosphate, and ascorbic acid. Adipogenesis protocols also utilize dexamethasone, in addition to isobutylmethylxanthine and indomethacin. Chondrogenesis protocols, on the other hand, typically utilize dexamethasone, ascorbic acid, sodium pyruvate, TGF-*β*1, and a combination of insulin-transferrin-selenium (ITS). However, variations of the components and their concentrations exist and the optimal formulations may depend on the subpopulation of MSC used and the ultimate therapeutic goal. MSCs predifferentiated toward these three lineages have been investigated extensively in the context of tissue engineering wherein cells are implanted at the site of desired repair or replacement, often along with a scaffold (Figure 1) [54–58].

Beyond the standard trilineage potential of MSCs, differentiation has also been observed toward other cell types, such as tenocytes, skeletal myocytes, cardiomyocytes, smooth muscle cells, and even neurons [59–61]. However, some of these claims have courted a degree of skepticism in regard to the frequency of differentiation and the functionality of the terminal cells produced, especially for nonmesenchymal and nonmesodermal cell types. For example, while MSCs have been shown to differentiate into neuron-like cells, the functionality of rat MSC-derived neurons has been called into question in terms of their capacity to generate normal action potentials [62, 63]. Similarly, human MSCs have also been reported to differentiate into endothelial-like cells; however, such cells have lower expression of endothelial markers compared to mature endothelial cells [64]. Further study into the differentiation frequency and normal functioning of MSC-derived terminally differentiated cells will be necessary, in addition to determining if different MSC populations are better suited to differentiate into some cell types than others. With regard to the latter, a recent study comparing human CD146^+^/CD34^−^/CD45^−^ MSCs isolated from different anatomical locations (bone marrow, periosteum, and skeletal muscle) revealed that each subpopulation differed considerably in their transcriptomic signature and *in vivo* differentiation potential, hence suggesting that MSCs are not a uniform population throughout the body [65]. Moreover, MSC heterogeneity may not only exist between tissue types but also within individual tissues. For example, locationally and transcriptionally distinct subpopulations of CD34^+^/CD146^−^ “adventitial MSCs” and CD34^−^/CD146^+^ “pericyte-like MSCs” have been found to reside in human adipose tissue, a commonly used cell source for regenerative medicine [66]. Similar findings have also been noted in horses and canines, suggesting these dual perivascular subpopulations are conserved in mammals [67, 68]. Interestingly, both equine and human adipose-derived CD34^−^/CD146^+^ MSCs display greater angiogenicity compared to CD34^+^/CD146^−^ MSCs indicative of a relatively conserved functional phenotype as well, possibly due to their pericyte-like differentiation state [67, 69]. Heterogeneity among MSCs may also have important implications for treating disease resulting from inappropriate differentiation and proliferation. Of note, subsets of PDGFR*β*^+^ and/or PDGFR*α*^+^ MSC-like progenitor cells with fibro-adipogenic potential have been found to be present in multiple tissues (*e.g.*, tendon, myocardium, and skeletal muscle) and may prove to be useful targets for reducing fibrotic damage after injury [70, 71]. Further investigation into MSC heterogeneity will be required to resolve if such differences are solely a result of innate differences arising from different developmental origins or if differing local microenvironments also play a role.

Unlike some other stem cell populations (e.g., hematopoietic stem cells), which have a well-established and relatively straight-forward unidirectional differentiation hierarchy, the hierarchy of MSC differentiation is currently poorly defined. To date, one of the MSC-like populations that have been most vigorously investigated in terms of hierarchy are human umbilical cord-perivascular cells (HUCPVCs). Such cells have been found to differentiate from quintipotential stem cells (with osteogenic, adipogenic, chondrogenic, myogenic, and fibrogenic potential) to a restricted fibroblast-state in a deterministic manner with a predictable order of loss in potency [72]. Whether this is true for all or some MSC populations remains to be examined, but this study should serve as a useful template for future investigation. As well, computational approaches that cluster cells according to differentially expressed genes may also help clarify the hierarchy of MSC subpopulations and their progeny cells [66] and may serve as a guide for future lineage tracing studies. That said, transdifferentiation toward nonmesodermal lineages and bidirectional phenotype switching between different mesenchymal cell types (e.g., transitions between fibroblasts and myofibroblasts or between synthetic and contractile smooth muscle cells) may further complicate any MSC hierarchical differentiation model established [73]. Regardless of any specific hierarchy and the potential for phenotypic plasticity, it should be emphasized that ultimately, the microenvironment dictates MSC behaviour, in terms of both their differentiation and their interaction with other cell types.

## 7. MSC Immunomodulatory Paracrine Signaling

Recently, a paradigm shift has occurred in the understanding of the therapeutic effects of MSCs. Despite the differentiation potential these cells exhibit and contrary to initial assumptions, in many therapeutic contexts, MSCs exert their healing effects not through engraftment and differentiation but rather through paracrine signaling and communication through cell-cell contacts [51, 74]. The significance of this paradigm change is reflected in the recent recommendation to rebrand MSCs as *medicinal signaling cells* by Arnold Caplan, who had originally coined the term mesenchymal stem cells [75]. Notable examples of MSC paracrine/juxtacrine-mediated treatments currently in preclinical and clinical development include injections into the myocardium after infarction, treatments for graft versus host disease (GvHD), and therapies for autoimmunity disorders (such as Crohn's disease and type I diabetes) [76–79]. Given these successes, it is becoming increasing clear that the MSC secretome has broadly beneficial effects that can be exploited for a wide range of therapeutic applications.

The MSC secretome contains a large range of molecules that are beneficial for tissue repair, including ligands that promote the proliferation and differentiation of other stem/progenitor cells, chemoattraction, antifibrosis, antiapoptosis, angiogenesis, and immunomodulation [80]. Currently, perhaps the most impactful of these properties from a clinical perspective is their capacity for immunomodulation, which has motivated the development of intravenous injections of MSCs, such as Osiris Therapeutics' Prochymal®, which is approved for GvHD in Canada and currently in clinical trials for several autoimmune disorders in Canada and the USA. This immunomodulatory capacity has been partly attributed to the ability of MSCs to inhibit effector T-cell activation and proliferation, both directly through various cytokines and indirectly through modulating the activity of regulatory T-cells [81, 82]. MSCs have also been described as modulating the behaviors of natural killer cells, dendritic cells, B-cells, neutrophils, and monocytes/macrophages through the actions of a number of molecules, including prostaglandin E2 (PGE2), indoleamine 2,3-dioxygenase (IDO), nitric oxide (NO), interleukin-10 (IL-10), and many others [8, 80]. Notably in the context of localized tissue repair, MSCs have been implicated in promoting alternative activation of macrophages toward a regenerative and proangiogenic M2 phenotype, as opposed to a classical proinflammatory M1 phenotype [83–86]. Consequently, given the many roles MSCs play in therapeutic immunomodulation and regeneration, it is becoming increasingly acknowledged that one of the main roles of adult MSCs *in vivo* may be to coordinate healing responses and to help prevent autoimmunity after injury [8, 74, 80].

Lastly, it should be noted that MSCs are not solely anti-inflammatory. Under certain conditions, MSCs can elicit an inflammatory response by presenting antigens to induce CD8^+^ T-cell responses, and increasing expression of MHCDII and presenting antigens to CD4^+^ T-cells [87–89]. The “switch” between eliciting an inflammatory or anti-inflammatory response generally seems to be whether the activating signals are associated with infections or tissue injury, respectively [90].

## 8. MSC Angiogenic Paracrine Signaling

In addition to being proangiogenic by promoting a regenerative microenvironment via immunomodulation, MSCs also directly secrete angiogenic factors that affect endothelial cell survival, proliferation, and migration. Such factors include key growth factors critical for initial vessel formation and subsequent stabilization, such as VEGF, FGF2, SDF1, ANG1, MCP-1, HGF, and many others [91, 92]. Beyond these classical angiogenic growth factors, MSCs also secrete microvesicles (>200 *μ*m) and exosomes (~50–200 *μ*m) that can carry both growth factors and miRNAs and have been demonstrated to have proangiogenic activities both *in vitro* and *in vivo* [93]. Such extracellular vesicles have been shown to enhance angiogenesis and healing in a number of contexts, including murine and rat models of burn injury, cutaneous wounds, myocardial infarction, and limb ischemia [94–99]. Recent proteomic analysis has found human MSC-derived exosomes contain a number of proteins associated with angiogenesis that were upregulated when MSCs were exposed to ischemic-like conditions, including PDGF, EGF, FGF, and NF-*κ*B pathway-affiliated proteins [100].

Similarly, recent qPCR screening of exosomes derived from murine MSC-like cells revealed they contain a number of known proangiogenic microRNAs, several of which were found to be preferentially internalized by endothelial cells, including miR-424, miR-30c, miR-30b, and let-7f [101]. Relatedly, miR-210 has also been implicated in the therapeutic effect of MSC-derived extracellular vesicles in a mouse model of cardiac infarction, as siRNA knockdown reduced the angiogenic effect of the vesicles [102]. Delineating which specific MSC-derived exosomal miRNAs are responsible for particular aspects of angiogenesis is an ongoing area of research. Recently, for example, exosomal miRNA-125a from human adipose-derived MSCs has been implicated in enhancing angiogenesis specifically by promoting tip cell formation through the inhibition of delta-like 4 (DLL4) [103]. Ultimately, however, as is the case with angiogenic growth factors, multiple miRNAs may have to work in concert to achieve maximal effects and interrogating which subsets are critical for different stages of angiogenesis will require further inquiry.

## 9. Direct Cellular Involvement of MSCs in Angiogenesis

In addition to their interaction via various paracrine routes, MSCs also participate in direct cell-cell contact with endothelial cells. When cocultured on or embedded within hydrogels (e.g., fibrin or Matrigel), endothelial cells form capillary-like structures on which MSCs may adhere and assume an abluminal position akin to their perivascular position *in vivo* [104]. This maintained mural cell behavior after culture may be exploited for microvascular tissue engineering as it has beneficial effects for the nascent endothelial tubules. For example, the permeability of these *in vitro* structures is decreased in the presence of MSCs relative to simply coculturing endothelial cells with fibroblasts potentially due to tighter cell-cell junctions and VE-cadherin expression [105]. This effect may also be attributed to increased basement membrane formation, as extensive studies of pericyte-endothelial cell cocultures have demonstrated that both the expression and deposition of basement membrane proteins is upregulated through cell-cell contact *in vitro* [106, 107]. However, any specific effects of MSCs on basement membrane formation and its composition, compared to non-multipotent pericytes, has yet to be elucidated.

Under *in vivo* contexts, MSCs can also assume a perivascular cell phenotype and have beneficial effects on vessel stability and permeability. For example, when collagen-fibronectin gels containing EGFP-labeled human MSCs and HUVEC (human umbilical vein endothelial cells) were implanted in cranial windows of SCID mice, implants with MSCs resulted in a higher vessel density compared to HUVEC-only implants, and EGFP colocalized with staining for the smooth muscle cell- (SMC-) related markers, *α*SMA and SM22*α* [108]. Similarly, when embedded within submillimeter collagen rods coated with endothelial cells and then implanted in an omental pouch within rats, GFP-labeled rat MSCs were found to migrate out of the modules and began to associate with blood vessels and express *α*SMA at day 7 postimplantation, while at day 21, all GFP^+^ MSCs were found to be in a perivascular position [109]. Strikingly, when examined by microCT after Microfil® injection, including MSCs within the implant created vasculature with reduced leakiness compared to endothelial cell-only controls which exhibited a leaky core.

Similarly, after subcutaneous injection of HUVEC and fibrin hydrogel into SCID mice, HUVEC-derived vessels formed after 7 and 14 days showed decreased permeability to 70 kDa dextran in conditions including human adipose and bone marrow-derived MSCs, compared to lung fibroblasts or endothelial cells alone [110]. Correspondingly, with this improved barrier function, only implants with ASCs and BMSCs contained vessels with abluminal calponin staining, suggestive of SMC differentiation of the implanted stromal cells. Collectively, it is clear that not only is the presence of a mesenchymal cell type advantageous for vessel formation and stabilization, but the identity of the mesenchymal cell type and its propensity to take on an abluminal position and perivascular cell phenotype has an impact on the functionality of the resulting vessels.

## 10. Clinical Considerations for Using Bone or Adipose MSC Sources

As noted previously, MSCs can be isolated from many different human tissues; however, the most common adult sources for clinical use are bone marrow and adipose tissue. This is due to a number of reasons, including the total cell numbers that can be harvested, the frequency of the cells of interest, and the relatively small procedural risk associated with obtaining cells from these locations compared to other anatomical locations. As well, in the case of adipose tissue removal, if the procedure is being carried out for other purposes (*e.g.*, elective cosmetic surgery), there is no additional risk associated with the harvesting of progenitor cells which would otherwise be discarded.

In the case of bone marrow aspirate, the procedure is generally carried out at the bedside using a local anesthetic (*e.g.*, lidocaine) with the posterior superior iliac spine being the preferred collection site owing to its relative ease of access [111]. After sterilization of the overlying skin, a fine gauge trocar is used to gain access to the marrow space, which then permits the subsequent aspiration of marrow by syringe [111]. For the purposes of stem cell harvesting, it is possible to harvest as much as 20 mL of marrow from a single aspirate site [112].

Bone marrow sampling is generally considered to be safe but can frequently result in pain during and after the procedure [113]. Preventative measures, such as first ensuring that the periosteum is adequately anesthetized, can be used to reduce the pain to acceptable levels [113]. Other adverse events during bone marrow sampling are rare, with an estimated event rate of 5/10,000 and a fatality rate of 1-2/100,000 [114]. In a 2013 survey conducted by the British Society of Haematology, out of a total of 19,259 bone marrow aspirates with or without trephine biopsies, clinically significant hemorrhage occurred in only 11 patients, while infections were seen in just two [114]. The risk of bleeding can be mitigated through careful patient selection and correction of underlying coagulopathies if necessary. When bleeding does occur, it is usually mild and can often be controlled by the manual application of pressure to the site [111]. In the event of more significant bleeding, arterial embolization has been demonstrated to be an effective hemostatic therapy [115]. The risk of infection can be mitigated by first ensuring an absence of any overlying skin or soft tissue infection or presence of osteomyelitis. In suspected occurrences of infectious complications, topical antimicrobials are generally considered to be adequate in most cases.

In contrast to bone marrow aspirate, adipose tissue—in the form of liquid fat from liposuction or solid fat from abdominoplasty—is obtained under general anesthetic with a greater risk of procedural morbidity and mortality [116]. In the case of liposuction, the targeted fat is removed via aspiration after injection of a sterile saline solution containing epinephrine and a topical anesthetic [116]. The process may be facilitated by the liquefaction of fat using ultrasound- or laser-assisted liposuction [116]. Conversely, abdominoplasty involves the surgical excision of excess solid adipose tissue and dermis.

Common adverse events for liposuction include postoperative nausea and vomiting, local nerve damage and paresthesias, intra- and postprocedural bleeding and hematomas, persistent edema, surgical wound infection, skin necrosis, and unplanned hospitalization or increased length of stay [117]. The risk of fatality of liposuction is conservatively estimated to be 1/5000 with deaths being attributable to pulmonary embolism, visceral perforation, cardiorespiratory complications associated with anesthesia, and hemorrhage (in order of decreasing frequency) [118]. Abdominoplasty is a more invasive procedure with higher rates of surgical complications, including wound dehiscence and necrosis, infection, and a fatality rate approaching 1/600 [119].

Given the relatively unfavorable risk profile associated with surgical collection of adipose tissue, the harvesting of adipose-derived MSCs is ideal for patients who are already planning on undergoing such a procedure. Otherwise, bone marrow aspirate remains a preferred option as it can permit the ad hoc collection of MSCs at a lower risk of morbidity and mortality. However, such clinical risks must be weighed against certain practical requirements as well.

In addition to considering the risks associated with the different anatomical sites and any contraindications specific to a certain patient, the preference for one tissue source over the other may also be affected by the number of desired cells that can be collected from a certain source and the quantity of cells requisite for a particular application. As summarized by Murphy et al., in the case of a bone marrow aspirate, approximately 109–664 CFU-F/mL can be obtained at a frequency of 10–83 CFU-F/10^6^ nucleated cells [120]. In contrast, lipoaspirate typically yields far more cells of interest per milliliter of tissue, with 2058–9650 CFU-F/mL at a frequency of 205–51,000 CFU-F/10^6^ nucleated cells [120]. Hence, if the quantity of cells that can be obtained via bone marrow aspirate are insufficient for a particular autologous application, relying on an adipose cell source instead may be a sensible option. This is especially true in situations where *ex vivo* culture must be limited to preserve a desired cellular phenotype or when culture is not utilized at all (*i.e.*, immediate autologous use of the stromal vascular fraction (SVF) after harvesting).

Beyond differences in the quantity of cells obtainable from either bone or adipose tissues, innate differences in differentiation ability between cell types may also affect the preference of one MSC population over the other for a particular application. Unsurprisingly, given their developmental and anatomical origins, adipose-derived MSCs have been demonstrated to have an increased capacity for *in vitro* adipogenic differentiation by Oil Red O staining, possibly due to their relatively higher expression of the adipogenesis-regulating transcription factor, PPAR-*γ*, after exposure to adipogenic stimuli [121, 122]. Similarly, bone marrow-derived MSCs have been demonstrated to have an increased capacity for osteogenic differentiation over MSCs derived from adipose tissue via alizarin red staining [121, 122]. This may be partly attributable to their higher expression of the key osteogenic transcription factor, Runx2, during osteogenic differentiation [122]. Moreover, bone marrow-derived MSCs have also been shown to have a higher capacity for chondrogenic differentiation (by alcian blue staining and collagen II expression), as may be expected considering the close relationship between chondrogenesis and osteogenesis in the generation of osseous tissues [121–123]. It should be noted, however, that some conflicting reports to these general findings also exist and suggest that whether adipose or bone-derived MSCs have the higher capacity to differentiate toward a particular lineage may depend on the characteristics of the patient (*e.g.*, sex, age, and disease state), the isolation protocol, and the differentiation conditions [90].

Other notable functional differences between the two cell types have been documented. For example, in a comparison of the immunomodulatory capacity of adipose and bone marrow-derived MSCs isolated from the same donor, Valencia et al. found that MSCs from bone marrow had a higher capacity to inhibit natural killer cytotoxic activity, whereas adipose-derived MSCs had a higher capacity to inhibit dendritic cell differentiation [124]. Corroborating this, other reports have also described similar findings regarding these differential effects on natural killer cells and dendritic cell differentiation [125, 126]. Similarly, differences in growth factor expression between the two cell types have also been noted and may influence which cell type to use in clinical applications where MSCs are intended to provide trophic support. For example, bone marrow-derived MSCs have been shown to produce significantly more HGF compared to adipose-derived MSCs, which may be an important consideration for regenerative therapies involving the liver [122]. Overall, the choice of bone or adipose sources is complex and is influenced by factors specific to the application and the patient. As the use of MSCs becomes increasingly common, the optimal choice of cell source for specific clinical circumstances will likely become clearer.

## 11. iPSC Sources and Epigenetic Reprogramming of MSCs

Despite the clinical promise of MSCs in allogeneic applications (or the use of HLA-matched donor cells), some therapies may necessitate an autologous approach, such as long-term implantation of MSC-derived engineered tissues. However, this presents a significant challenge in cases where the desired cell type cannot be obtained in sufficient numbers to be clinically useful. This may occur in the case of needing to engineer particularly large replacement tissues, as MSCs have limited expansion capability in culture, partly due to their low to absent expression of telomerase [127, 128]. As well, this may occur when patients have insufficient MSCs of adequate quality due to age or disease. With regard to aging, CFU-F frequency within the bone marrow generally declines with age, and the capacity of the remaining MSCs to withstand oxidative stress appears to also decline along with their function and therapeutic efficacy [129–131]. Such functional changes may be the result of progressively shortening telomeres, accumulated molecular damage, and stochastic genetic and epigenetic changes over time [132–136]. Such age-associated epigenetic dysregulation may also contribute to alterations in the differentiation potential and heterogeneity of MSCs [137, 138]. In addition to age-induced functional decline, conditions such as type 2 diabetes and metabolic syndrome may similarly limit the therapeutic potential of MSCs for autologous use due to increased oxidative stress, mitochondrial dysfunction, and increased senescence [139].

One potential solution to address this issue is iPSC (induced pluripotent stem cell) technology in which somatic cells from a patient are first reprogrammed to a pluripotent state, usually by the overexpression of transcription factors (*e.g.*, KLF4, c-MYC, OCT4, and SOX2) [140, 141]. Favorably, such cells can then be expanded *in vitro* extensively prior to differentiation, partly due to their expression of telomerase. Also favorably, especially for cells harvested from aged patients, once harvested cells are differentiated into the desired cell type after having been in a transient pluripotent state results in longer telomeres compared to the starting donor cell along with a “rejuvenated” epigenetic landscape with reduced aging-associated epigenetic marks and increased resistance to oxidative stress [142, 143].

Multiple studies have explored methods for differentiating MSC-like cells from iPSCs [143–149]. These reports have described iPSC-MSCs as being largely comparable to mature MSCs in terms of trilineage potential, immunomodulation, and trophic support. However, some minor differences have also been noted, such as differences in adipose differentiation, T-cell regulation, sensitivity to NK cells, and their expression levels of certain genes (*e.g.*, interleukin-1 and TGF*β* receptors) [143, 150–153]. Interestingly, a recent study by Chin et al. reported that differentiation of human pluripotent stem cells into MSCs results in two distinct subpopulations with different trophic phenotypes [149]. One subpopulation with higher expression of CD146 and CD73 could maintain HSCs (hematopoietic stem cells) *ex vivo* and expressed HSC niche-related genes, while a second subpopulation with lower expression of CD146 and CD73 displayed poor maintenance of HSCs. Such *in vitro* findings using iPSCs are intriguingly reminiscent of *in vivo* MSC heterogeneity and may not only help provide a source of MSCs for clinical use but may also help elucidate the developmental origins of different MSC subpopulations.

While iPSCs are a promising source of MSCs, they do carry the risk of malignant transformation during culture and teratoma formation after transplantation due to residual pluripotent cells [154]. Alternative means for returning aged or diseased MSCs to a more therapeutically effective state without relying on a transient pluripotent stage may also exist. One option may consist of using the pluripotency genes used for creating iPSCs but for a shorter duration in order to elicit partial reprogramming and reverse age-associated epigenetic marks but not loss of cellular identity. Such an approach yielded impressive results in mice in terms of improving recovery from metabolic disease and increasing muscle regeneration after transient *in vivo* overexpression [155]. It remains to be seen, however, if this approach has a beneficial effect on MSCs as well. Future work will need to focus on determining the optimal dosing regimen for human cells and examining if this method is useful for the *ex vivo* rejuvenation of human MSCs.

Alternatives for rejuvenating cells that do not rely on pluripotency genes at all also exist, which may be preferable for further mitigating tumorigenic risks. One option may be to alter the levels of beneficial or detrimental miRNAs within cultured MSCs prior to transplantation. For example, Okada et al. unveiled that miR-195 plays a key role in inducing senescence in murine bone marrow-derived MSCs by inhibiting the expression of telomerase [156]. When the authors inhibited miR-195, telomere lengths and cellular proliferation were increased compared to control cells. Most importantly though, using a mouse model of acute myocardial infarction, the authors demonstrated that when transplanted the rejuvenated cells resulted in reduced infarct size and improved left-ventricle function.

Conversely, upregulation of certain molecules, such as miR-543 and miR-590-3p may also be useful in preventing senescence given their inhibitory roles in senescence onset in MSCs [157]. Upregulation of SIRT1, a NAD^+^-dependent deacetylase, has also been shown to prevent MSC senescence possibly through increasing telomerase activity and reducing DNA damage [158]. Strikingly, overexpression of telomerase and myocardin in aged murine MSCs resulted in improved therapeutic efficacy when used in a model of hindlimb ischemia, in terms of stimulating arteriogenesis and increasing blood flow [159]. Regardless of whether particular factors are upregulated or downregulated, it should be stressed that any approach that alters regulators of senescence, telomere length, and/or pluripotency will require extensive investigation in order to ensure that rejuvenation of MSCs does not come at the cost of increasing tumorigenesis.

## 12. Clinical Risks and Challenges

As of May 2018, there are currently 82 active and recruiting trials involving “mesenchymal stem cells” listed by ClinicalTrials.gov in the United States alone, in addition to 44 already completed studies; moreover, there are also 27 active/recruiting trials involving “mesenchymal stromal cells” with 9 already completed. Of these ongoing studies, the majority are currently in phase 1 followed by phase 2 trials. Given these appreciable number of trials and their early stages, it will be crucial to discern if any patterns of adverse effects can be detected among MSC clinical trials in order to develop effective solutions to these issues. The risks involved in these trials are partly dependent on the route of administration of MSCs (Figure 1).

In terms of risks involving the systemic infusion of MSCs, Lalu et al. conducted a meta-analysis of clinical trials with both autologous and allogeneic MSCs and concluded that this route of administration appears generally safe as their analysis did not find any significant association between MSC infusion and acute toxicity, infection, organ system complications, malignancy, or death [160]. There was, however, a significant association with transient fever in some patients. Other studies have also identified chill, infection, and liver damage as potential adverse effects of systemic administration [161, 162]. Lalu et al. also commented on the frequent absence of reporting follow-up duration for long-term adverse events in the studies they examined and noted that it is critical that future studies investigate both short-term and long-term adverse events given that experimental cell-based therapies may have serious long-term consequences (*e.g.*, immunological complications, causing/enhancing neoplastic growth). Favorably for risk mitigation, however, there is evidence to suggest that MSCs that are infused systemically generally do not persist over the long-term [163]. Also favorably, of the 13 studies examined by Lalu et al. that used unmatched allogeneic MSCs, none reported acute infusional toxicity. Such findings bode well for systemically administered therapies requiring large quantities of cells that cannot be acquired from a single patient and for cases in which a patient's own MSCs may be functionally inadequate and/or inaccessible due to underlying disease.

Regardless, while MSCs themselves appear generally safe for systemic infusion, biological and chemical components associated with the *ex vivo* culture and storage of MSCs, such as fetal bovine serum (FBS) and dimethylsulfoxide (DMSO) may introduce risks in the clinical use of MSCs. Such components warrant caution due to the possibility of infectious contamination, immunogenicity, and/or infusional toxicity [164, 165]. With regard to zoonotic concerns regarding FBS, such risks may be addressed through the use of human platelet lysate in place of FBS for supporting the *ex vivo* growth of MSCs [164, 166].

Risks and their associated challenges regarding more experimental interventions involving the local injection of MSCs and implantation of engineered tissues are currently less well defined compared to the more commonly used systemic administration route. Currently, challenges associated with these approaches often relate to first establishing clinically significant efficacy in order to justify these more invasive procedures. Some key challenges for local injections include maintaining cell viability, increasing MSC permanence after injection, and optimizing delivery to a specific location [161, 167, 168]. In regard to this, rapidly gelling injectable hydrogels have shown promise in targeting MSCs to specific anatomical locations and in maintaining their viability after injection to prolong therapeutic function [169]. Currently, investigations into generating engineered tissues are primarily focused on ensuring comparable function to native tissues (or at least, similar enough to be therapeutically useful). Key challenges include optimizing the differentiation process, developing effective scaffold materials, and ensuring sufficient maturation of the nascent tissues through chemical and mechanical cues [73, 170, 171]. As well, depending on the tissue type and its dimensions, the issue of vascularization either pre- or postimplantation must also be addressed in order to preserve function and to avoid ischemia-induced inflammation. As discussed previously in this review, MSCs themselves may be of use in this regard given their proangiogenic signaling and native perivascular phenotype. Ostensibly, some engineered tissues may be optimally composed of MSC-derived terminally differentiated cells along with angiogenic undifferentiated MSCs, in order to fully take advantage of both their differentiation and angiogenic capabilities.

## 13. Concluding Remarks

Efforts into understanding and exploiting MSCs for therapeutic use have garnered a multifaceted view into the capabilities of these cells, albeit sometimes in a nonlinear and even serendipitous manner. Contrary to many other clinical successes for drugs and cell therapies alike, where a comprehensive understanding of the therapeutic mechanism(s) is first established before being employed clinically, MSCs have had remarkable successes despite a limited understanding of their *in vivo* function under normal physiological conditions. To further improve and build on these early successes, future work will need to be directed toward understanding the more nuanced aspects of these cells. As alluded to earlier, this will partly involve developing an improved understanding of the differences between MSCs found in different anatomical locations and the heterogeneity that exists within these subpopulations, in addition to performing rigorous investigation into the functional differences between cells differentiated from MSCs and native terminal cells. By building on the body of MSC research that has been produced thus far, potential risks in downstream clinical applications can be mitigated and the therapeutic potential of MSCs may be further expanded upon to benefit patients in a wide range of clinical settings.

## Figures and Tables

**Table 1:** Potential markers for MSC identification and enrichment.

Selection type (and comments)	CD No.	Name	Acronym	Reference
Negative	**CD11b**	**Integrin subunit alpha M**	**ITGAM**	[5]
Negative	**CD14**	**CD14 molecule**	**CD14**	[5]
Negative	**CD19**	**CD19 molecule**	**CD19**	[5]
Negative (not in all MSC populations)	**CD34**	**CD34 molecule**	**CD34**	[5]
Negative	**CD45**	**Protein tyrosine phosphatase, receptor type C**	**PTPRC**	[5]
Negative	**CD79a**	**CD79a molecule**	**CD79A**	[5]
Negative (unless stimulated with IFN-*γ*)	**—**	**Human leukocyte antigen, antigen D Related**	**HLA-DR**	[5]
Positive	CD9	CD9 molecule	CD9	[172]
Positive	CD10	Membrane metalloendopeptidase	MME	[173]
Positive	CD13	Alanyl aminopeptidase, membrane	ANPEP	[174]
Positive	CD29	Integrin subunit beta 1	ITGB1	[175]
Positive	CD44	CD44 molecule (Indian blood group)	CD44	[176]
Positive	CD49f	Integrin subunit alpha 6	ITGA6	[177]
Positive	CD54	Intercellular adhesion molecule 1	ICAM1	[178]
Positive	CD71	Transferrin receptor	TFRC	[179]
Positive	**CD73**	**5**′**-nucleotidase ecto**	**NT5E**	[5]
Positive	**CD90**	**Thy-1 cell surface antigen**	**THY1**	[5]
Positive	**CD105**	**Endoglin**	**ENG**	[5]
Positive	CD106	Vascular cell adhesion molecule 1	VCAM1	[11]
Positive	CD146	Melanoma cell adhesion molecule	MCAM	[10]
Positive	CD166	Activated leukocyte cell adhesion molecule	ALCAM	[180]
Positive	CD200	CD200 molecule	CD200	[181]
Positive	CD271	Nerve growth factor receptor	NGFR	[12]
Positive	CD349	Frizzled class receptor 9	FZD9	[173]
Positive	CD362	Syndecan 2	SDC2	[182]
Positive (a disialoganglioside, nonpeptide)	—	Ganglioside GD2	G2	[183]
Positive (also known as nucleostemin)	—	G protein nucleolar 3	GNL3	[184]
Positive (target of anti-STRO1 antibodies)	—	Heat shock protein family A (Hsp70) member 8	HSPA8	[185]
Positive	—	Heat shock protein 90 beta family member 1	HSP90B1	[186]
Positive (a glycosphingolipid, nonpeptide)	—	Stage-specific embryonic antigen-4	SSEA-4	[187]
Positive	—	Sushi domain containing 2	SUSD2	[188]
Positive	—	Alkaline phosphatase, liver/bone/kidney	ALPL	[13]

Bolded text indicates markers recommended by the International Society for Cellular Therapy (ISCT) for minimally defining human multipotent mesenchymal stromal cells by positive and negative selection.
